# ToxPi*GIS Toolkit: creating, viewing, and sharing integrative visualizations for geospatial data using ArcGIS

**DOI:** 10.1038/s41370-022-00433-w

**Published:** 2022-04-26

**Authors:** Jonathon Fleming, Skylar W. Marvel, Stacy Supak, Alison A. Motsinger-Reif, David M. Reif

**Affiliations:** 1 Bioinformatics Research Center, Department of Biological Sciences, North Carolina State University, Raleigh, NC USA; 2 Center for Geospatial Analytics, College of Natural Resources, North Carolina State University, Raleigh, NC USA; 3 Biostatistics and Computational Biology Branch, National Institute of Environmental Health Sciences, National Institutes of Health, Durham, NC USA

**Keywords:** Visual analytics, Geographic information systems, Data integration

## Abstract

**Background:**

Presenting a comprehensive picture of geographic data comprising multiple factors is an inherently integrative undertaking. Visualizing such data in an interactive form is essential for public sharing and geographic information systems (GIS) analysis. The Toxicological Prioritization Index (ToxPi) framework offers a visual analytic integrating data that is compatible with geographic data. ArcGIS is a predominant geospatial software available for presenting and communicating geographic data, yet to our knowledge there is no methodology for integrating ToxPi profiles into ArcGIS maps.

**Objective:**

We introduce an actively developed suite of software, the ToxPi*GIS Toolkit, for creating, viewing, sharing, and analyzing interactive ToxPi profiles in ArcGIS to allow for new GIS analysis and an avenue for providing geospatial results to the public.

**Methods:**

The ToxPi*GIS Toolkit is a collection of methods for creating interactive feature layers that contain ToxPi profiles. It currently includes an ArcGIS Toolbox (*ToxPiToolbox.tbx*) for drawing location-specific ToxPi profiles in a single feature layer, a collection of modular Python scripts that create predesigned layer files containing ToxPi feature layers from the command line, and a collection of Python routines for useful data manipulation and preprocessing. We present workflows documenting ToxPi feature layer creation, sharing, and embedding for both novice and advanced users looking for additional customizability.

**Results:**

Map visualizations created with the ToxPi*GIS Toolkit can be made freely available on public URLs, allowing users without ArcGIS Pro access or expertise to view and interact with them. Novice users with ArcGIS Pro access can create *de novo* custom maps, and advanced users can exploit additional customization options. The ArcGIS Toolbox provides a simple means for generating ToxPi feature layers. We illustrate its usage with current COVID-19 data to compare drivers of pandemic vulnerability in counties across the United States.

**Significance:**

The integration of ToxPi profiles with ArcGIS provides new avenues for geospatial analysis, visualization, and public sharing of multi-factor data. This allows for comparison of data across a region, which can support decisions that help address issues such as disease prevention, environmental health, natural disaster prevention, chemical risk, and many others. Development of new features, which will advance the interests of the scientific community in many fields, is ongoing for the ToxPi*GIS Toolkit, which can be accessed from www.toxpi.org.

## Introduction

Geographic data are used to support decisions and inform analysis at many scales, from neighborhoods and larger communities to states and nations. Combined with sophisticated geographic information system (GIS) tools, these data can be a powerful means of communicating issues, and their drivers, that are affecting communities and can act as a catalyst for change. Because of the complexity of places and their underlying environments, data from a single source is often insufficient for accurately communicating a geographical area’s issues and the drivers of problems such as health disparities. To remedy this, summary quantitative layers, or scores, can be created by aggregating multiple data sources [1, 2]. While these layers or scores convey the combined effect of numerous factors on a population, there is typically no mechanism to convey the contributions of individual factors to the overall score. For a truly comprehensive picture of geographic data comprising multiple factors, visual analytics are needed that present both integrated scores and the contributions of the distinct factors driving these scores.

**Fig. 1:**
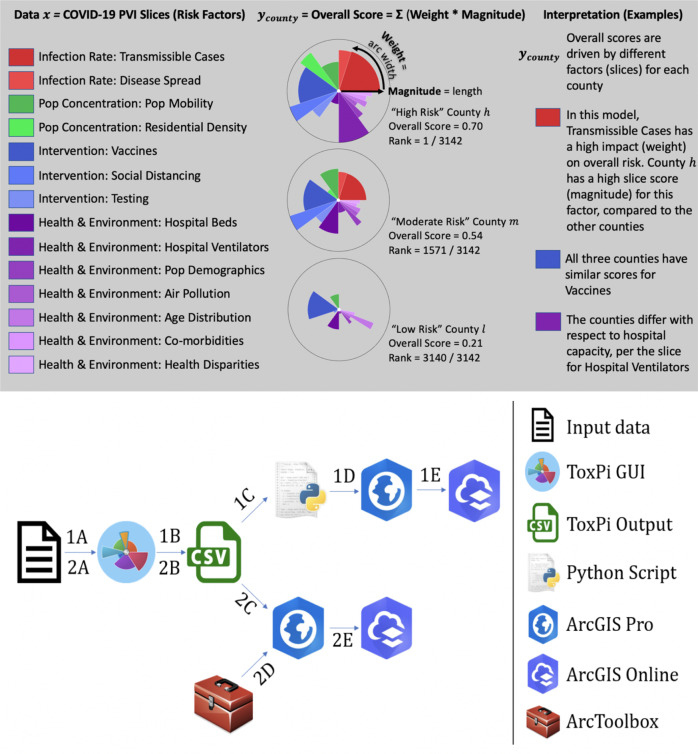
Summary of the ToxPi model used and the Toolkit workflow options. (Top) Overview of the ToxPi framework for COVID-19 vulnerability in the new ‘PVI with Vaccine Model’ with four categories (color families) grouped into 14 slices of data. (Bottom) Schematic of the ToxPi*GIS Toolkit workflow Methods 1 and 2, from raw data to map sharing.

One such integrative visual analytic is the Toxicological Prioritization Index (ToxPi) framework, which is a statistical framework for aggregating data from various sources. The framework accepts continuous and ordinal data for integration as sets of related, user-defined factors into slice-wise and overall scores [3]. These scores are displayed together as a ToxPi profile, which are variants of polar diagrams or radar charts [4], wherein each “slice” integrates information from one or more data sources into the corresponding factor score (see Fig. 1). The profiles provide a visual summary of each factor’s weighted contribution to the overall score. The framework can flexibly incorporate whatever combination(s) of data the user chooses, and the typical model is built so that a higher score translates to higher risk/concern/vulnerability/etc. The ToxPi framework has been used with a wide range of data types with many different outcomes of interest, including decision support and hazard assessment by bodies such as the International Agency for Research on Cancer and the National Academy of Sciences [5–7], environmental impact due to natural disasters [8, 9], data-driven clustering for substances of unknown/variable composition [10], and clinical phenotype evaluation [11]. Models can be built with a user-friendly ToxPi GUI (Graphical User Interface) application [12] or the *toxpiR* package (https://CRAN.R-project.org/package=toxpiR). Both model-building options generate output that is compatible with the ToxPi*GIS Toolkit introduced here.

In a GIS context, the ToxPi framework provides an integrative model layer atop geospatial data, for a compact summary of the factors driving differences in analysis results among regions. Geospatially, it has been deployed within custom-built dashboards created to assess vulnerability to COVID-19 at the county level across the United States [13], to compare hurricane susceptibility at the census-tract level in the greater Houston area [14], and to visualize the interplay of stressors related to children’s environmental health across North Carolina [15]. The ToxPi*GIS web application has also been used in tandem with ArcGIS tools/dashboards for parallel analysis [16]. These studies have demonstrated the utility of graphics that integrate location-specific multivariate data for addressing the disparate effects of various drivers on vulnerability and susceptibility in different locations. They have shown that ToxPi deployed within a geospatial context can be an effective means of communication to guide community adaptation, reprioritization, and change. However, these studies use either static maps for ToxPi profiles, applications that are not publicly editable, or results that do not take full advantage of powerful GIS capabilities. Integration of ToxPi profiles within ArcGIS maps would address these issues, providing ToxPi users a framework for geospatial analysis, mapping, and interactive public sharing with any dataset and model.

ArcGIS Pro is an advanced GIS software that provides extensive capabilities for geospatial data analysis and map production, and ArcGIS Online provides a user-friendly platform from which Web Maps and Web Mapping Applications can be created to share data and information. Despite extensive capabilities, so far, ArcGIS does not provide a tool for creating ToxPi profiles as ArcGIS data layers. To address this issue, we developed an ArcGIS Pro Toolbox that draws location-specific ToxPi profiles as feature layers for placement on an ArcGIS map. The use of ArcGIS tools often requires preliminary data manipulation and several sequential, often complicated geoprocessing steps to generate new information, making geospatial problem solving a daunting task for inexperienced ArcGIS users. To simplify map creation of location-specific ToxPi profiles, we developed a custom Python script, *ToxPi_creation.py*, that handles all data preparation, generates a layer file containing the drawn ToxPi profiles, and displays the layer based on the user’s choices in the ToxPi GUI. We combined the toolbox and the Python script to create the ToxPi*GIS Toolkit, a continuously developing repository of software for integrating ToxPi statistics and the results of geospatial analysis.

The applications are detailed for those without ArcGIS Pro access, novice users with ArcGIS Pro access but limited expertise, and advanced users looking to create customized, integrated ArcGIS workflows and distributable dashboards. With the help of the documentation and vignettes, novice users of ArcGIS Pro can generate shareable maps with limited knowledge of the software, and advanced users can use the Toolkit to generate ToxPi feature layers with options for customization, integration, and distribution. Created maps can be made freely available on public URLs so that ArcGIS Pro access is not required to view and interact with them. Here, we illustrate applications of the ToxPi*GIS Toolkit using publicly available COVID-19 data to compare the pandemic vulnerability of counties across the United States. All applications, usage instructions, sample data, example visualizations, and open-source code are freely available from a dedicated GitHub page linked on www.toxpi.org.

## Methods

The ToxPi*GIS Toolkit is an addendum of methods to be used alongside ToxPi GUI, a free, platform-independent Java application for recombining diverse source data into ToxPi profiles (www.toxpi.org). The toolbox methods proposed in this paper can be used to produce interactive feature layers (groupings of similar geographic features and associated properties) containing ToxPi profiles for map creation and visualization.

### Toxicological Priority Index (ToxPi)

The ToxPi framework provides a method for transparently integrating and visualizing data from multiple information sources. These information sources are often of disparate types, measurement units, and/or distributions. ToxPi recombines data into a dimensionless index that is the overall “score” for a given data record, which for geospatial data is tied to a specific location. The overall score is comprised of weighted slices, where each slice is a recombination of one or more vectors of source information (see Fig. 1).

The calculations in ToxPi are detailed in [3, 17] and, and open-source code is available from the *toxpiR* package (https://CRAN.R-project.org/package=toxpiR). Briefly, scores compress all data into a [0-1] interval, where 0 represents the minimum observed value and 1 represents the highest observed value for a given slice. The slice scores are then renormalized into a [0-1] interval of overall scores, where 1 again represents the highest observed score for a given record. In the illustrative example used to demonstrate the ToxPi*GIS Toolkit, each data record (e.g., Wake County, NC), has related vectors of information (e.g., demographic data on diabetes and obesity) grouped into slices (e.g., “Co-morbidities”) representing factors contributing to high overall scores (e.g., vulnerability to COVID-19). In this example, slice-wise scores represent a given county’s risk profile—relative to all other counties—for each factor. The combination of slice-wise scores into overall scores represent a given county’s overall risk profile—relative to all other counties—for all factors combined. The conventional interpretation is that higher scores translate to higher risk/vulnerability/hazard, so part of model formulation is specifying how numeric values in source data are scaled. For source data such as infection rates, higher values directly correspond to higher risk. For source data where lower values correspond to higher risk (e.g., percentage of a given population without insurance), users should specify that the “raw” source numeric values be inverted so that higher ToxPi scores can be interpreted as higher risk. Thus, when visualizing scores as ToxPi profiles (see Fig. 1), the intuition is maintained that “longer slice means higher risk” for factor slices and “more filled area means higher vulnerability” for overall scores.

### ToxPi*GIS Toolkit

The ToxPi*GIS Toolkit uses custom Python scripts combined with ArcGIS Pro methods and a custom ArcGIS Pro Toolbox to generate feature layers that contain ToxPi profiles drawn at the location to which the data are tied, enabling the comparison of results across a region. These feature layers can be loaded onto a new Pro Map or Web Map with existing data, enabling new comparisons. Furthermore, each slice is drawn as a separate polygon, which allows users to select an individual slice to obtain more information about the location it represents. These details are displayed in popups that can be easily reconfigured to display important, underlying data used in the analysis. The ToxPi*GIS Toolkit currently includes two suggested methods for generating these feature layers, which are described below. The pipelines for the use cases are outlined at the bottom of Fig. 1.

### Method 1- Creation of ToxPi feature layers using Python scripts

Method 1 consists of a Python script, *ToxPi_creation.py*, that can be run from the Windows command prompt. The output of ToxPi GUI is used as input to produce ToxPi feature layers. The script automates all geoprocessing and data manipulation steps required for ToxPi feature layer production and has only two required input parameters—the location of the input data file and the location for the script output. The script also provides an optional parameter to scale the size of ToxPi profiles so users can make adjustments based on their data’s scope and density, as well as an optional parameter to provide a boundary layer based on the data’s unity of analysis (e.g., county boundaries).

The simplicity of this method allows users of all experience level to quickly and easily produce feature layers that are ready to be shared as a map. These feature layers are saved to a layer file (.lyrx) that can be opened with ArcGIS Pro and shared to ArcGIS Online for public viewing. Users can create a shareable map by opening an output layer file in ArcGIS Pro and selecting the option to publicly share it to ArcGIS Online, allowing anyone with the URL to view and interact with the map. A map owner can also allow users to copy and further analyze the map and its underlying data to produce new, interactive visualizations.

This method is suggested for users who are unfamiliar with ArcGIS Pro as well as those who do not require significant customization and want to bypass manual data and feature class preparation. The script’s input-data formatting requirements are outlined in the Toolkit documentation, and an evolving Utilities folder contains information that can help with these requirements.

### Method 2- Creation of ToxPi feature layers using an ArcGIS Toolbox

**Fig. 2:**
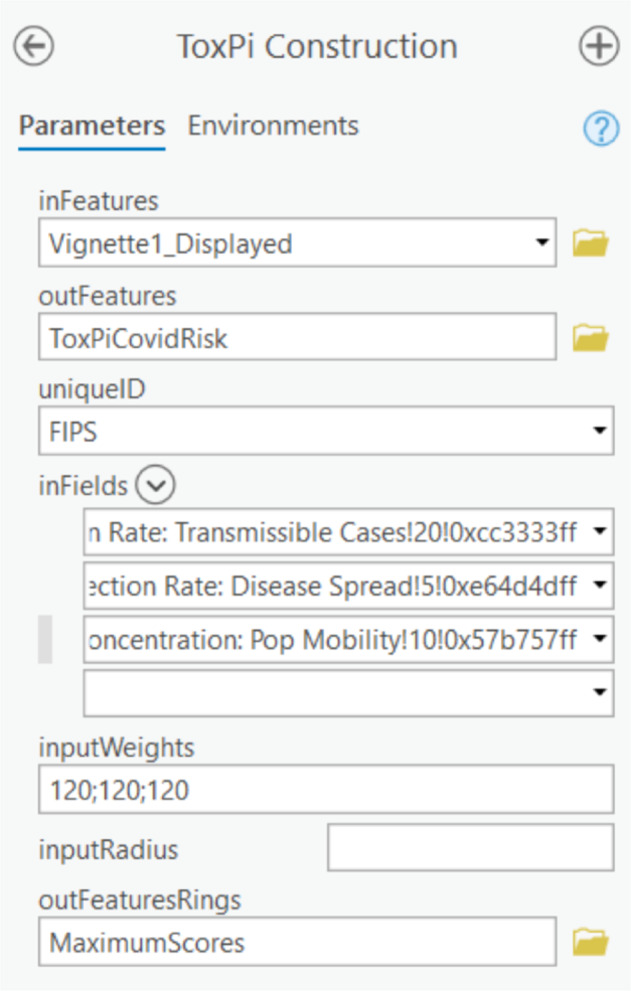
Interface for ToxPi Construction using the ToxPi Toolbox. Parameters: inFeatures - the name of the input feature layer; outFeatures – the name of the output feature class; uniqueID – the unique identifier for each data point; inFields – A list of the slices to include in the ToxPi profiles; inputWeights – A string of weights that correspond to the list of fields for determining radial width; inputRadius – optional, value to scale ToxPi size by; outFeaturesRings – optional, name of the maximum radius rings feature layer.

**Fig. 3:**
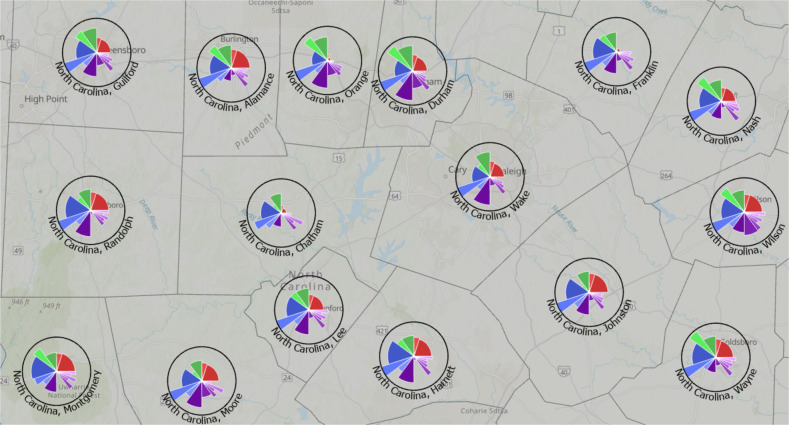
Output of *ToxPi_creation.py* for COVID-19 vulnerability data zoomed in on a central region of North Carolina. The boundary layer shown represents county boundaries in North Carolina and was obtained by setting the optional boundary parameter of the script to county. Labels (optional) indicate the state and county of each profile.

Method 2 consists of an ArcGIS Pro Toolbox, *ToxPiToolbox.tbx*. This method produces a feature layer that can be integrated into other analysis pipelines and shared to ArcGIS Online for public viewing. While this method allows more customization of feature layers than the Python script, including the ability to draw a subset of slices and use different coordinate systems, it requires more data preparation and manual geoprocessing, which the script automates. The Toolkit documentation contains an example walkthrough of the steps for data preparation and the creation of ToxPi feature layers. While experienced ArcGIS Pro users can easily customize some layers during preparation, including associating more underlying data with each ToxPi profile, several steps are compulsory for preparing toolbox input data. Notably, feature classes or layers prepared for the tool must be converted into a projected coordinate system prior to input. Figure 2 depicts the ToxPi Construction geoprocessing tool which includes parameters that must be set for the tool to function. Figure 3 provides example output produced by running the python script or toolbox with COVID-19 data, from which local vulnerability to COVID-19 can be compared.

### Advancement and versatility of ToxPi_creation.py

**Fig. 4:**
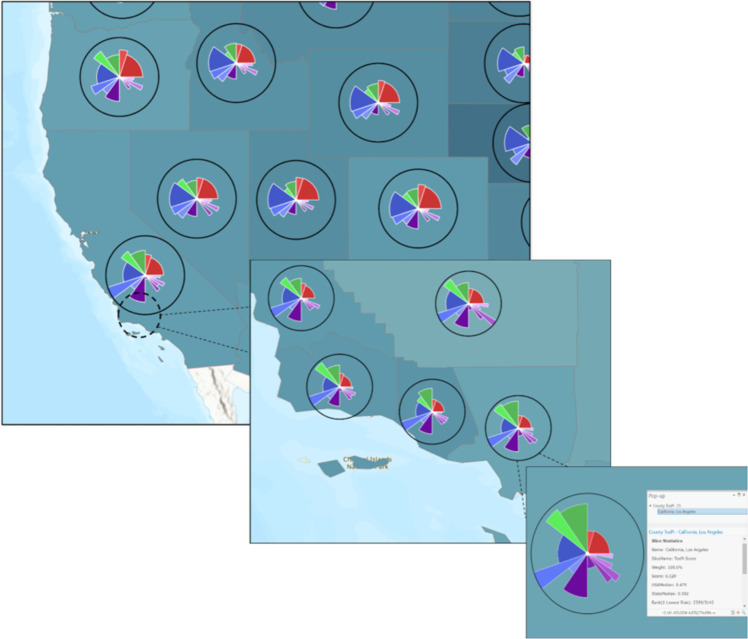
Output of *ToxPi_creation_customized.py* with COVID-19 vulnerability data showing the switching of feature layers based on zoom extent. The top left (zoomed out) is a state median feature layer, the middle (zoomed in) is a county ToxPi feature layer, and the bottom right (zoomed in further) is the scrollable information pop-up when selecting that particular locality.

To demonstrate the versatility of the ToxPi*GIS Toolkit and the customization capacity provided by ArcGIS, we added advanced geoprocessing steps and analysis for use with county- or census-tract-level data to the *ToxPi_creation.py* script for Method 1. The resulting script, *ToxPi_creation_customized.py*, can be run with the same workflow as Method 1 but produces more advanced maps for a specific geographic data type. This method generates extra layers for visualization, including two different-sized layers of ToxPi profiles and choropleths, which can be useful if the end-users want to view the data at different zoom levels (i.e., the smaller feature layer can be displayed when comparing census tracts and the large feature layer can be displayed with comparing different states). Figure 4 contains static images of a map at different geospatial extents that was produced by running the altered script with COVID-19 vulnerability data, thus showing the multilayer capability of the altered script and demonstrating the integration of ToxPi_creation.py with other advanced geoprocessing steps.

### Data collection

We obtained data for the COVID-19 vulnerability map from the Pandemic Vulnerability Index (PVI) Dashboard at https://covid19pvi.niehs.nih.gov/. The PVI Dashboard provides two existing models for data analysis, one of which contains vaccine data while the other does not. The data used as an example in this paper was analyzed using the model containing vaccine data and was collected on June 24th, 2021. In this model, risk components are split into four categories—infection rate, population concentration, intervention measures, and health and environment—that comprise 14 categories. Figure 1 shows each category’s corresponding weight. This model accounts for vaccine data in the intervention measures domain, allowing for a more accurate representation of COVID-19 risk than previous models. More information about the model, including the underlying components for each slice category, can be found at the National Institutes of Health (NIH) PVI Dashboard details page (https://www.niehs.nih.gov/research/programs/coronavirus/covid19pvi/details/). We used these data to demonstrate the extended capabilities of the ToxPi*GIS Toolkit, including its integration with powerful analysis methods available in ArcGIS and the visualization capabilities of ArcGIS Online.

### New advanced analysis options provided by ArcGIS Pro

To demonstrate how county-level results from the COVID-19 vulnerability model created with *ToxPi_creation_customized.py* can be further used in knowledge generation, we performed hotspot analysis, a powerful statistical analysis method offered in ArcGIS Pro, using ToxPi scores. We conducted the hotspot analysis in ArcGIS Pro using the Optimized Hotspot Analysis tool with a distance band of 50 miles. Given incident points or weighted features (points or polygons), this tool creates a map of statistically significant hotspots and coldspots using the Getis-Ord Gi* statistic and evaluates the input feature class to determine if they are statistically significantly clustered or dispersed. The null hypothesis for this tool and this data is that the features themselves are completely spatially random. The z-scores and p-values returned by the tool determine whether that null hypothesis can be rejected or not. If the z-score and *p*-value indicate that the null hypothesis can be rejected with a particular confidence, then the features exhibit statistically significant clustering or dispersion. The Optimized Hotspot Analysis output is z-score and *p*-value for each feature within the input feature class [18]. In this case, each county was placed into a Gi_Bin based on the confidence level that it was clustered in a hotspot or coldspot according to the ToxPi score. By default, seven bins were generated, +/−3, +/−2, +/−1, and 0, where + denotes a hotspot, − demotes a cold spot, 3 denotes a 99% confidence interval, 2 denotes a 95% confidence interval, 1 denotes a 90% confidence interval, and 0 denotes no statistical significance.

**Fig. 5:**
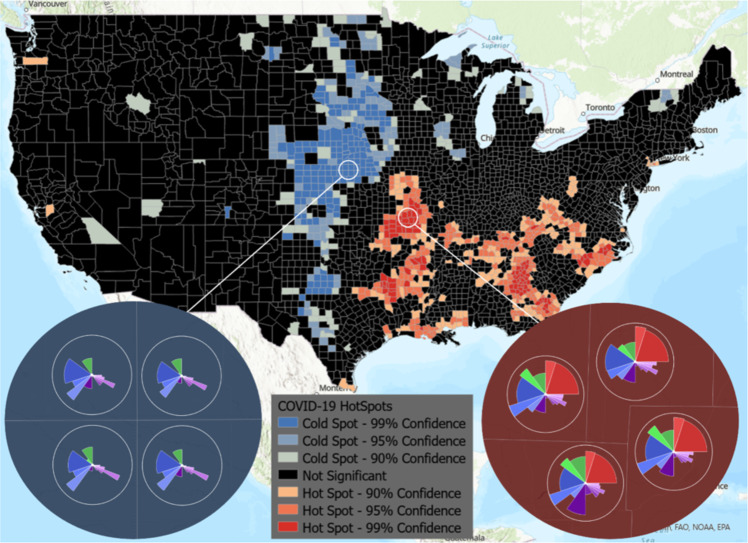
COVID-19 vulnerability hotspot analysis based on ToxPi Score. A national view of hotspots and coldspots is shown, where color represents clustering of high-risk scores (hotspot) and clustering of low-risk scores (coldspot). The two insets show ToxPi profiles for example coldspot (blue) and hotspot (red) counties with a 99% confidence level, providing an easy means of visually comparing the areas based on slice scores.

**Fig. 6:**
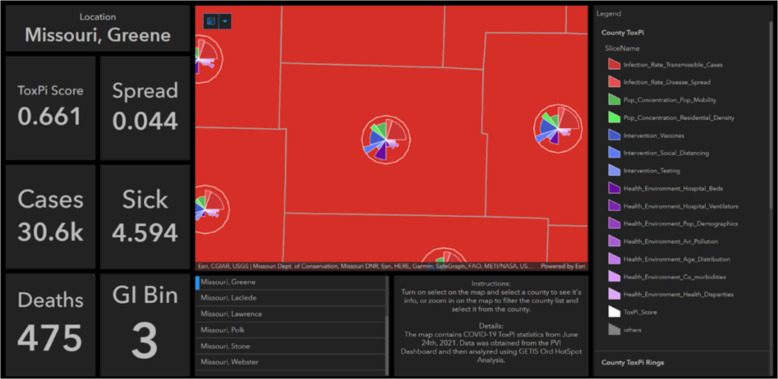
Example dashboard. This example dashboard was created by importing the hot spot analysis results joined with the raw data into an ArcGIS Online Dashboard and configuring panels to show important statistics for each county. Selecting a county will give its ToxPi score, spread and sick score, number of cases and deaths, and the Gi_Bin it was placed in to determine its significance as a hot or cold spot.

As shown in Fig. 5, the choropleth layer output can be used to see where the z-scores are large and confidence is high (statistically significant hotspots shown in red) and where the z-scores are small and confidence is high (statistically significant coldspots shown in blue). Zooming in on this layer displays the ToxPi profiles, which can be used as a visual reference for further analysis of the factors causing these clusters. Although the analysis was conducted using the overall ToxPi score, similar analysis could be done using a specific slice to allow for individual factor’s hotspots/coldspots to be compared across a geographic region. To demonstrate some of the powerful hosting and visualization capabilities ArcGIS provides, we joined the hotspot analysis results with raw data and displayed the result in an ArcGIS dashboard for public viewing (Fig. 6).

## Results

All interactive maps and visualizations are linked from www.toxpi.org to the dedicated Github page. Figure 3 is a static image, zoomed in on a portion of North Carolina, of the model created with COVID-19 data using *ToxPi_creation.py*. This method results in an interactive feature layer of ToxPi profiles that visually matches the results of *ToxPiToolbox.tbx* (Note that minor visualization differences/options are described in the Toolkit documentation). Analyzing the map, it can be seen that at the time of data collection, vulnerability differences between neighboring counties were driven by vaccination rates, availability of hospital beds, and infection rates. These drivers suggest prioritization of resources toward hospitals and vaccination efforts.

Figure 4 is a static image of the result of the model created by *ToxPi_creation_customized.py* with COVID-19 data. The figure shows local to regional to state displays that aggregate data by user-selected zoom extent. Because of the interactive nature of these profiles, users can select individual slices to learn more about the risk factors they represent, enabling quick analysis of the factors driving COVID-19 risk at different geographic scales of interest.

Figure 5 is a static image of hotspot analysis results and shows that cold spots are found in Nebraska and the edges of South Dakota and Kansas. Hotspots are primarily found in Missouri, the border of Texas, Arkansas, Georgia, and Tennessee. The zoomed-in views show the ToxPi feature layer for an example hotspot and a coldspot, allowing direct visual comparison of the different factors affecting the regions. The ToxPi profiles clearly show that transmissible cases, disease spread, residential density, social distancing, population demographics, and health disparities are highly different between the hotspot and coldspot.

Figure 6 is a static image of hotspot analysis results displayed on a publicly viewable dashboard. Important statistics can be easily retrieved for a county of interest. The flexible customization of this powerful visualization technique enables users to tell a story about a location based on a dataset. The figure shows COVID-19 statistics, including cases, deaths, sickness scores, and disease spread rate for Greene County, Missouri, a major hotspot.

## Discussion

We developed two new methods using ArcGIS Pro to create feature layers containing ToxPi profiles. The first method comprises a Python script, *ToxPi_creation.py*, that is user-friendly and suitable for ArcGIS users of all experience levels as well as those who want to produce a feature layer containing only ToxPi profiles. The script automates the required geoprocessing steps, so after loading the ArcGIS Pro environment, users simply run the script with the results of the ToxPi GUI or *toxpiR* applications as input. Further, as the script uses the underlying code for the toolbox, users are not required to download and add the toolbox to the proper environment, making it especially accessible for inexperienced ArcGIS users.

Because the script is open source, experienced Python users can easily access both the toolbox code and the code used for data preparation and thus develop new related methods and analysis procedures. To demonstrate how potential users can alter *ToxPi_creation.py, we* present the *ToxPi_creation_customized.py* script that uses both geoprocessing procedures and Python analysis to create advanced maps. The modifications to this script support county- and census-tract-level input data and support additional ArcGIS Hotspot analysis. The flexibility afforded by our development efforts will provide new opportunities for multi-factor visualization to be used in decision support for many fields.

The second method comprises an ArcGIS Pro Toolbox, *ToxPiToolbox.tbx*, and is recommended for advanced ArcGIS users due to the geoprocessing steps required for data preparation. The method allows customization of the output feature layer and can be easily integrated into existing ArcGIS workflows or models. As there is no current method for generating ToxPi feature layers within ArcGIS, the contribution of this toolbox seeks to advance the options for visualizing and exploring multifactor data within their locational context.

For either method path (i.e., python script or ArcGIS toolbox) or customization level, users can tailor the models themselves using either the ToxPi GUI or *toxpiR* package. This includes options for data apportionment into single-source slices (i.e., more slices) versus aggregated slices (i.e., fewer slices), slice weighting, coloration, and other model parameters. Thus, users can create as many ToxPi models as needed for thorough analysis, then use the ToxPi*GIS Toolkit to place selected models into geospatial context.

The choice of COVID-19 data as an example use case of this script is a relatable demonstration and a useful visualization of data of interest to the general public. For example, results from the Optimized Hotspot Analysis tool in ArcGIS revealed that significant high-risk clusters are located in Missouri, the border of Texas, Arkansas, Georgia, and Tennessee. Combined with the ToxPi*GIS Toolkit, this method enables the easy visualization of factors affecting high-risk areas. The results can help support decisions regarding how resources should be allocated across a specific set of counties to address the health disparities contributing to their vulnerability to COVID-19. Although the results displayed here use past data for a rapidly changing topic, the intention was to demonstrate a topical use case, rich in public data, for how the ToxPi*GIS Toolkit can help users explore score data, gain insight into the data leading to those scores within a geographical context, and couple this exploration with additional ArcGIS analysis methods.

There are several avenues available in the ArcGIS universe to make further use of ToxPi*GIS Toolkit output. ArcGIS dashboards represent one of many powerful ArcGIS Online visualization tools for conveying information that portray the story behind data. A dashboard offers many advantages over a stand-alone web map, namely customization and the ability to include additional analytics within a single interface. Another communication option offered by ArcGIS would be to incorporate ToxPi into a story map, which would support viewers taking a “tour” including walking interactively through an analysis and key findings.

The ToxPi*GIS Toolkit, as well as instructions for its use, are freely available on a dedicated GitHub page. Development of this living software Toolkit will continue, with the objective of developing new methods that integrate ToxPi statistics. We provide open-source code to enable users to create new methods, with the objective of advancing the interests of the scientific community. All example visualizations and the dedicated GitHub page are linked at www.toxpi.org, which will be regularly updated with advancements related to ToxPi methodology and applications. Future goals include updates to provide additional options for scaling and filtering ToxPi profiles, automate methods for separating overlapping ToxPi profiles, and add the ability to integrate time-scale data.

## Supplementary information

Supplementary Information

## Data Availability

All applications, usage instructions, sample data, example visualizations, and open-source code are freely available from a dedicated GitHub page linked from www.toxpi.org. ArcGIS Pro can be obtained at https://www.esri.com/en-us/arcgis/products/arcgis-pro/overview.
