# Supplementary material for: ToxPi*GIS Toolkit: creating, viewing, and sharing integrative visualizations for geospatial data using ArcGIS
Source: J Expo Sci Environ Epidemiol. 2022 Apr 26;32(6):900–7. doi: 10.1038/s41370-022-00433-w (PMC9039976; doi:10.1038/s41370-022-00433-w)
Supplement: Supplementary file 1 — Supplementary Information [file 41370_2022_433_MOESM1_ESM.docx]

**SUPPLEMENTAL and OTHER OPTIONAL MATERIAL**

All applications, usage instructions, sample data, example visualizations, and open-source code are freely available from a dedicated GitHub page linked from [www.toxpi.org](http://www.toxpi.org). Direct links to specific elements described above are provided here.

ToxPi*GIS Toolkit Github

<https://github.com/Jonathon-Fleming/ToxPi-GIS>

Vignette1: *ToxPi_creation.py* Demonstration

<https://ncsu.maps.arcgis.com/home/item.html?id=7c0365b3f75949369b46c07ae4ecf10c>

Vignette2: *ToxPi_creation_customized.py* Demonstration

<https://ncsu.maps.arcgis.com/home/item.html?id=1518637a0b454036a3d0d2fc8239ff08>

HotSpot/Dashboard Demonstration

<https://ncsu.maps.arcgis.com/home/item.html?id=022416cbc74d430691ad7d2a4cbec229>
